# Selection and characterisation of a phage-displayed human antibody (Fab) reactive to the lung resistance-related major vault protein

**DOI:** 10.1038/sj.bjc.6600159

**Published:** 2002-03-18

**Authors:** G L Scheffer, A W Reurs, B Jutten, S H W Beiboer, R van Amerongen, M Schoester, E A C Wiemer, H R Hoogenboom, R J Scheper

**Affiliations:** Department of Pathology, Free University Medical Center, De Boelelaan 1117, 1081 HV, Amsterdam, The Netherlands; Department of Pathology, Maastricht University, Maastricht, The Netherlands; Institute of Hematology, Erasmus University, Rotterdam, The Netherlands

**Keywords:** LRP, MVP, multidrug resistance, Fab, phage display.

## Abstract

The major vault protein is the main component on multimeric vault particles, that are likely to play an essential role in normal cell physiology and to be associated with multidrug resistance of tumour cells. In order to unravel the function of vaults and their putative contribution to multidrug resistance, specific antibodies are invaluable tools. Until now, only conventional major vault protein-reactive murine monoclonal antibodies have been generated, that are most suitable for immunohistochemical analyses. The phage display method allows for selection of human antibody fragments with potential use in clinical applications. Furthermore, cDNA sequences encoding selected antibody fragments are readily identified, facilitating various molecular targeting approaches. In order to obtain such human Fab fragments recognising major vault protein we used a large non-immunized human Fab fragment phage library. Phages displaying major vault protein-reactive Fabs were obtained through several rounds of selection on major vault protein-coated immunotubes and subsequent amplification in TG1 *E coli* bacteria. Eventually, one major vault protein-reactive clone was selected and further examined. The anti-major vault protein Fab was found suitable for immunohistochemical and Western blot analysis of tumour cell lines and human tissues. BIAcore analysis showed that the binding affinity of the major vault protein-reactive clone almost equalled that of the murine anti-major vault protein Mabs. The cDNA sequence of this human Fab may be exploited to generate an intrabody for major vault protein-knock out studies. Thus, this human Fab fragment should provide a valuable tool in elucidating the contribution(s) of major vault protein/vaults to normal physiology and cellular drug resistance mechanisms.

*British Journal of Cancer* (2002) **86**, 954–962. DOI: 10.1038/sj/bjc/6600159 www.bjcancer.com

© 2002 Cancer Research UK

## 

Multidrug resistance (MDR; reviewed in Schneider *et al*, 1999) is a phenomenon frequently observed in tumour cells that are unresponsive to anti-cancer drugs. Proteins which can be involved in this process are the ATP-binding Cassette (ABC) transporter proteins (Higgins, 1992) P-glycoprotein (P-gp, ABCB1; reviewed in Ambudkar *et al*, 1999), multidrug resistance protein 1 (MRP1, ABCC1), MRP2 (ABCC2), MRP3 (ABCC3; reviewed in Borst *et al*, 2000) and breast cancer resistance protein (BCRP, ABCG2; Doyle *et al*, 1998), as well as the lung resistance-related protein (LRP; Scheffer *et al*, 2000). LRP was identified as the major vault protein (MVP), the main component of vaults (Scheffer *et al*, 1995).

Vaults are large multimeric barrel-shaped particles that are highly conserved through evolution, and likely to play an essential role in normal cell physiology. They are composed of multiple copies of three proteins (240, 193 and 104 kDa in size) and unique untranslated RNA species. The 104 kDa subunit is termed the MVP as it constitutes >70% of the particle mass. The p240 minor vault protein is identical to the telomerase-associated protein (TEP1) (Kickhoefer *et al*, 1999a), a structural protein in the telomerase complex. The other high molecular weight minor vault protein, p193, is a poly (ADP-ribose) polymerase (PARP) and is therefore named VPARP, for vault-PARP (Kickhoefer *et al*, 1999b).

The hollow structure of vaults is consistent with a role in either the compartmentalisation of macromolecular assemblies or in subcellular transport. In multidrug resistant tumour cells up-regulation of human *LRP/MVP* mRNA and protein as well as vault particle copy number was observed (Scheper *et al*, 1993; Laurencot *et al*, 1997; Kickhoefer *et al*, 1998). Primary evidence for a direct causal relationship between *LRP/MVP* expression and drug resistance was provided by Kitazono *et al* (1999). They showed that reduction of *LRP/MVP* expression by use of LRP/MVP-specific ribozymes in a cell line induced to overexpress *LRP/MVP* by exposing cells to sodium butyrate was enough to prevent drug resistance. Still, these studies have to be confirmed by independent investigations.

In order to further study the function of vaults and their contribution to MDR, specific antibodies are essential. Several MVP-reactive murine monoclonal antibodies (Mabs) are now available. These Mabs were all produced by classical hybridoma technology. In the past years the technology of display on filamentous phage in combination with selection was developed into a powerful tool for the identification of antibodies of human origin (Marks *et al*, 1991; Winter *et al*, 1994; Hoogenboom *et al*, 1998). Partially or completely human antibodies are of particular interest as they are expected to elicit no or minimal immune responses when administered to patients. For the construction of antibody libraries, V-genes are amplified from B-cell cDNA, and heavy and light chain genes are randomly combined and cloned to encode a combinatorial library of single-chain variable fragments (scFv) or Fab antibody fragments (Clackson *et al*, 1991; Hoogenboom *et al*, 1991). Large non-immune human antibody libraries, made for example from the variable region genes of the B-cells of healthy individuals, have been used to select antibodies to a large panel of antigens, including self-, non-immunogenic- and relatively toxic antigens (Marks *et al*, 1991; de Haard *et al*, 1999). When using the phage display system, the cDNA of antibodies of interest becomes available without further cloning steps. In this study we used a large non-immunised human Fab fragment phage library described by de Haard *et al* (1999) to isolate a human Fab reactive to the MVP.

## MATERIALS AND METHODS

### *Escherichia coli* strain

*Escherichia coli* TG1: K12, D(*lac-pro*), *supE*, *thi*, *hsd*D5/F' *tra*D36, *pro*A^+^B^+^, *lacl*^q^, *lac*ZDM15.

### Selection of phages displaying anti-MVP human Fab

For the selection of phages expressing anti-MVP Fab fragments a large non-immunised human Fab fragment phage library was used which was described by de Haard *et al* (1999). The library contains cDNA sequences, encoding highly diverse Fab fragments (3.7×10^10^) with additional c-*myc* and His tags, cloned in the phagemid vector pCES1.

Selection of phages was essentially according to published methods (Marks *et al*, 1991) on immunotubes (Nunc maxisorp; Life Technologies, Merelbeke, Belgium) coated overnight at 37°C with 20 μg recombinant full-length MVP (Schroeijers *et al*, 2000) in 2 ml coating buffer (0.05 M sodium carbonate, pH 9.6). Unbound antigen was removed and the tubes were blocked for 1 h with PBS/2% skimmed milk powder/0.05% Tween 20 (MT–PBS). One ml of the page stock solution containing approximately 1×10^13^ phages was diluted 1 : 1 in MT–PBS and allowed to bind to the antigen-coated immunotube for 2 h at room temperature. Unbound phages were washed off by 20 washes with PBS/0.05% Tween 20 followed by 20 washes with PBS. Antigen-bound phages were eluted off, using 1 ml freshly prepared 100 mM triethylamine (TEA) for 10 min. The solution was neutralised by adding 0.5 ml of Tris buffer (1 M, pH 7.4). The solution was incubated for another hour at room temperature on immunotubes coated only with block buffer, to remove aspecifically bound phages. Unbound, antigen-specific phages were used to infect exponentially growing (OD_600_ of 0.5) TG1 bacteria, for 30 min at 37°C. After infection, the bacteria were allowed to grow overnight at 30°C and glycerol stocks were made. To have an indication of the phage input/output ratio, titrations of the bacteria, immediately after infection, were plated on selective 2×TY (16 g l^−1^ trypton, 10 g l^−1^ yeast extract and 5 g l^−1^ NaCl) plates supplemented with 2% glucose and 100 μg ml^−1^ ampicillin. The plates were incubated overnight at 30°C and colonies were counted the following day. For the next selection round, a sufficient amount of bacteria to ensure the presence of at least 10 bacteria of each clone, was inoculated in 50 ml 2×TY medium containing 100 μg ml^−1^ ampicillin and 2% glucose, giving a start OD_600_ of approximately 0.05. Five ml of bacteria grown at 37°C to an OD_600_ of 0.5 were infected with 2 μl M13-K07 helper phage (approximately 1×10^13^ phages per ml) for 30 min at 37°C. The bacteria were spun down, resuspended in 50 ml of 2×TY medium containing 100 μg ml^−1^ ampicillin and 25 μg ml^−1^ kanamycin to select for super-infected bacteria, and grown overnight at 30°C. The bacteria were spun down, the selected phages secreted into the supernatant were precipitated twice using 1/5th volume 20% PEG 6000; 2.5 M NaCl, resuspended in 1 ml of PBS and used in the next selection round on antigen coated immunotubes. A total of four, essentially identical selection rounds was carried out on MVP-coated immunotubes.

### Screening of individual phage clones

To select individual phage clones displaying MVP-reactive Fabs, single bacterial colonies were grown at small-scale overnight at 30°C in 2×TY/2% glucose/100 μg ml^−1^ ampicillin in U-shaped 96-well plates (Costar, Corning, NY, USA). These plates were kept as ‘master plates’ at −80°C after adding glycerol to a final concentration of 15%. Then, 1 : 100 dilutions of the bacterial colonies were grown for 2 h at 37°C in V-shaped 96-well plates (Costar). Phages displaying recombinant Fab were produced by adding 25 μl of 1 : 250 diluted helper phage stock solution. Supernatants containing phages were tested for reactivity in recombinant MVP coated ELISA plates. ELISA plates were coated overnight at 37°C with 100 μl, 5 μg MVP per ml coating buffer (0.05 M sodium carbonate, pH 9.6). Plates were rinsed, blocked with 200 μl PBS/1% BSA/0.05% Tween 20 and incubated with phage-containing supernatant for 1 h at room temperature. Bound phages were detected using an in-house produced guinea pig-anti-phage polyclonal antiserum (1 : 1000) and HRP-labelled swine–anti-guinea pig antiserum (1 : 500, Dako, Copenhagen, Denmark). Colour development was with 5-amino-2-hydroxybenzoic acid (5-AS; Merck, Darmstadt, Germany) and 0.02% H_2_O_2_ as a chromogen.

### Large-scale production of Fab fragments

Large-scale induction of soluble Fabs from individual clones was performed on a 50 ml scale in 2×TY medium, containing 100 μg ml^−1^ ampicillin and 2% glucose. The bacteria were grown at 37°C to an OD_600_ of 0.9. The cells were pelleted, resuspended in 50 ml 2×TY, containing 100 μg ml^−1^ ampicillin and 1 mM isopropyl-1-thio-β-D-galactopyranoside (IPTG) to activate the *LacZ* promoter, and grown for 4 h at 30°C. Soluble Fabs were purified making use of the His-tag on Ni-NTA-superflow agarose beads according to the manufacturer's protocol (Qiagen Inc., CA, USA).

### *Bst*NI fingerprinting

Individual bacterial colonies were used for standard 40 cycle hot-start PCR reactions, using fd-tet-seq24 (5′-TTT GTC GTC TTT CCA GAC GTT AGT-3′) as a forward and M13-reverse (5′-AGC GGA TAA CAA TTT CAC ACA GG-3′) as a reverse primer. The resulting amplimers were digested with *Bst*NI and loaded onto a 2% agarose gel. The banding pattern of the clones was analysed on a UV transluminator.

### Sequence analysis

Plasmids from individual bacterial clones were sequenced using the dideoxy sequencing method on a semi-automated sequencer (Alf Express, Pharmacia, Uppsala, Sweden). CH1 for (5′-GTC CTT GAC CAG GCA GCC CAG GGC-3′) and M13 reverse (5′-AGC GGA TAA CAA TTT CAC ACA GG-3′) were used as forward and reverse primers, respectively. V-gene sequences were aligned to V-BASE (http://www.mrc-cpe.cam.ac.uk/imt-doc/public/INTRO.html).

### Immunoprecipitation

Cells were trypsinised and lysed in PBS containing 1% NP40, 1 mM EDTA, 1 mM phenylmethylsulphonyl fluoride (PMSF) for 2 min at room temperature, followed by a boost with an ultra-sound finger. Large fragments and nuclei were removed by centrifugation at 9000 *g*. Clear supernatant was pre-incubated for 30 min with protein A-sepharose beads to reduce non-specific binding of labelled protein. Cell lysate supernatants or solutions containing approximately 10 μg recombinant MVP were incubated with 10 μl soluble Fab or, as a control, 10 μg of LRP-56 MAb 2 h at room temperature. To the Fab precipitation 100 μl of anti-c-*myc* mouse monoclonal antibody 9E10 was added and incubation was continued for another hour. Then 50 μl of prot A-sepharose beads (∼25 μl ‘packed beads’) was added and precipitation was allowed for 1 h. Precipitates were washed three times in lysis buffer and three times in PBS. Antibody–antigen interaction was broken by dispensing the beads in Western blot loading buffer, containing 200 mM Tris-HCl (pH 6.8), 1% β-mercaptoethanol, 8% SDS, 10% glycerol and 0.05% bromophenol blue.

### Western blot analysis

Total cell lysates were made as described (Zaman *et al*, 1994). Protein concentrations were determined with a BioRad protein assay (BioRad, Richmond, CA, USA). Cell lysates (10–40 μg) or 2 μg recombinant MVP or immunoprecipitates were fractionated on a 8% polyacrylamide slab gel and transferred onto a nitrocellulose membrane by electroblotting. After blocking, the membrane was incubated for 2 h with anti-MVP Fab in an appropriate dilution. HRP-labelled rabbit–anti-human lambda chain (1 : 200, Dako) was used as a secondary antibody. Colour development was with 0.5 g l^−1^ 3,3′-diaminobenzidine tetrahydrochloride, 0.15 g l^−1^ chloronaphthol and 0.02% H_2_O_2_ in PBS.

### BIAcore analysis

The affinity of the anti-MVP antibodies was further examined by surface plasmon resonance on a BIAcore 2000 instrument (Biacore AB, Uppsala, Sweden). Recombinant fusion protein of MVP and glutathione-S-transferase (GST–MVP; Schroeijers *et al*, 2000) was immobilised on the flow cell of an activated CM-5 sensorchip according to the manufacturer's protocol (Biacore AB), yielding a surface of 2000 resonance units. Soluble Fab fragments and control murine Mabs were analysed at room temperature, at a flow rate of 25 μl per minute, using HBS-EP (Biacore AB, 0.01 M HEPES, pH 7.4, 0.15 M NaCl, 3 mM EDTA, 0.005% polysorbate 20) as a buffer. Dissociation rates were calculated from the sensorgrams using the BIA-evaluation software (Biacore AB).

### Immunohistochemistry

Cytospin preparations and cryosections (4 μm) were air dried overnight and fixed at room temperature for 7 min in 100% acetone or for 10 min in 3% paraformaldehyde/0.3% glucose. For detection of MVP with MVP-37 Mab and human Fab MVP-φ4, the slides were pretreated with 6 N guanidine hydrochloride Fab MVP-φ4, the slides were pretreated with 6 N guanidine hydrochloride (GdnHCl) in 50 mM Tris-HCl (pH 7.5) for 10 min at room temperature, as described (Schroeijers *et al*, 2000). The slides were incubated with primary antibody for 1 h at room temperature or at 37°C for the GdHCl treated slides. Biotinylated rabbit-anti-mouse (1 : 150, Zymed, San Francisco, CA, USA) and HRP-labelled streptavidin (1 : 500, Zymed) were used as secondary reagents for the mouse Mabs. For detection of the human Fab, 1 : 10 diluted anti-c-*myc*-tag 9E10 Mab, and HRP-labelled rabbit-anti-mouse serum (1 : 500, Dako, Copenhagen, Denmark) was used.

Colour development was with 0.4 mg ml^−1^ amino-ethyl-carbazole (AEC) and 0.02% H_2_O_2_ as a chromogen.

### Cell lines

The lung cancer cell lines SW1573 (parent), SW1573/2R120 (MVP and MRP1 positive), SW1573/2R160 (MDR1 P-gp positive and a low percentage of MVP positive cells) were described in reference (Kuiper *et al*, 1990). The lung cancer cell lines GLC4 (parent) and GLC4/ADR (MVP and MRP1 positive) were described in reference (Zijlstra *et al*, 1987). All cell lines were grown in Dulbecco's modified essential medium (Gibco Europe, Paisley, Scotland), supplemented with 10% heat-inactivated foetal calf serum, 2 mM L-glutamine, penicillin and streptomycin. Resistant cell lines were cultured in the presence of drugs until 3–10 days before the experiments. All cells were negative for *Mycoplasma* as tested by the Gene-Probe rapid Mycoplasma detection system (Gene-Probe, San Diego, CA, USA).

### Antibodies

Murine anti-MVP Mabs used were LRP-56 (Scheper *et al*, 1993), LMR-5 (Flens *et al*, 1997), MVP-9, MVP-16, MVP-37 (Schroeijers *et al*, 2001) and LRP (Transduction Laboratories, Lexington, KY, USA).

The anti-c-*myc* mouse Mab 9E10 was described in Chan *et al* (1987). The guinea pig-anti-phage polyclonal antiserum was produced by immunizing guinea pigs with wild type M13-K07 helper phages suspended in Freund's complete adjuvant (Difco, Detroit, MI, USA). Two booster injections with phages in PBS were given. Serum was collected and used without further purification.

## RESULTS

### Selection of anti-MVP human Fab

The successive rounds to select anti-MVP human Fabs on immunotubes coated with recombinant MVP, showed a gradual increase in the amount of antigen bound phages (output number). When the output number of round one was set to 1, output numbers of 50, 500 and 10 000 were noted for round 2, 3 and 4, respectively (Table 1

**Table 1:**
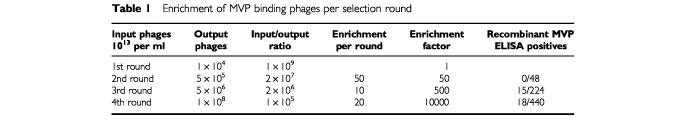
Enrichment of MVP binding phages per selection round

). Apparently, the cycles of selection and re-amplication yielded increasing numbers of antigen-binding phages. ELISA screening of approximately 750 individual clones from round 2, 3 and 4, resulted in the identification of several MVP-reactive phages. The percentage of antigen binding phages per selection round is depicted in Table 1. Twenty-two individual MVP-reactive clones from round 3 and 4 were selected for *Bst*NI fingerprinting. These clones all showed a very similar banding pattern, that was strikingly different from a randomly selected non-reactive clone (Figure 1

**Figure 1:**
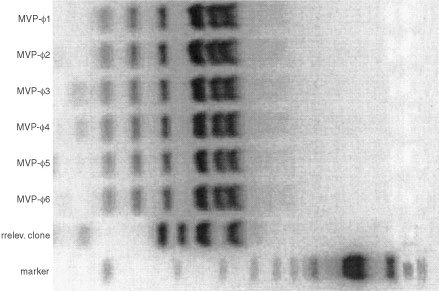
*Bst*NI fingerprinting of six MVP-reactive clones from selection rounds 3 and 4 and a randomly selected non-reactive clone. cDNA of individual clones was PCR amplified, *Bst*NI digested and loaded on an agarose gel. The MVP-reactive clones all show a very similar banding pattern, strikingly different from the irrelevant clone.

). Sequence analysis of six of these clones confirmed that they were all identical. One clone, named MVP-φ4, was used for further characterization studies. The sequences of the heavy and light chain variable regions of the MVP-φ4 clone are available under accession numbers AJ306838 and AJ306837, respectively, at the EMBL database.

### Heavy and light chain usage of the anti-MVP Fab

**Figure 2:**
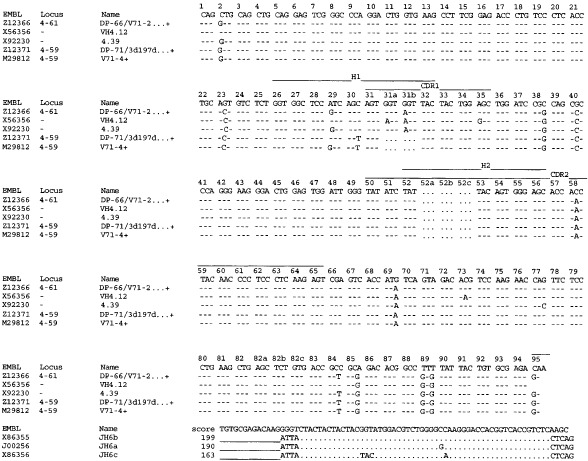
Comparison of the obtained heavy chain cDNA sequence of the anti-MVP Fab MVP-φ4 (accession number AJ306838) with V-BASE. The Fab uses a heavy chain belonging to the VH4 family. Highest homology was observed to germline clone DP-66/V71-2…+. The antigen specific CDR3 sequences for the heavy chain are: QGVYYYYGMDV. The number of amino acid changes from the germline, excluding the CDR3 region, is eight.

Comparison of the obtained cDNA sequence of the anti-MVP Fab with V-BASE showed that the Fab uses a heavy chain belonging to the VH4 family and a light chain belonging to the VL6 family (Figures 2

and 3

**Figure 3:**
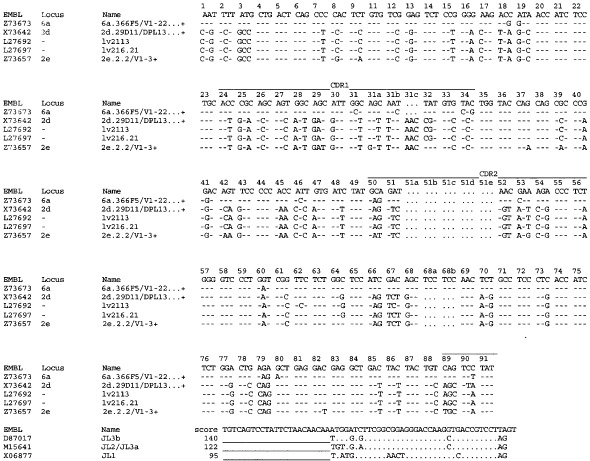
Comparison of the obtained light chain cDNA sequence of the anti-MVP Fab MVP-φ4 (accession number AJ306837) with V-BASE. The Fab uses a light chain belonging to the VL6 family. Highest homology was observed to germline clone 6a.366F5/V1-22…+. The antigen specific CDR3 sequences for the light chain are: QSYSNNKWI. The number of amino acid changes from the germline, excluding the CDR3 region, is 10.

). Highest homology was observed to germline clones DP-66/V71–2…+ (heavy chain) and 6a.366F5/V1–22…+ (light chain). The antigen specific CDR3 sequences for the heavy and light chain are: QGVYYYYGMDV and QSYSNNKWI, respectively. The number of amino acid changes from the germline, excluding the CDR3 region, is eight for the heavy chain and 10 for the light chain.

### Reactivity of anti-MVP Fab in Western blots and immunoprecipitation

**Figure 4:**
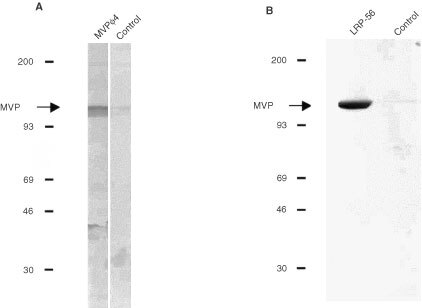
Western blots of cell lysates of *MVP*-overexpressing GLC4 ADR cells (**A**) and the immunoprecipitation products of the anti-MVP mouse Mab LRP-56 (**B**; left lane) or a control Mab (**B**; right lane) from a cell lysate of the GLC4 ADR cells. Proteins were loaded, separated and blotted. Blots were incubated with MVP-φ4 (**A**, left lane and **B**) or a control Fab (**A**, right lane) followed by HRP-labelled rabbit-anti-human lambda chain. Colour development was with DAB/chloronaphthol/H_2_O_2_. On both blots, MVP-φ4 was reactive with the MVP protein at the expected M_*r*_ of 110 000 (arrow).

In Western blots of cell lysates of *MVP*-overexpressing GLC4 ADR cells, MVP-φ4 was reactive with the MVP protein at the expected M_*r*_ of 110 000 (Figure 4A

). Furthermore, MVP-φ4 was able to detect the M_*r*_110 000 immunoprecipitation product of the anti-MVP mouse Mab LRP-56 and a cell lysate of the GLC4 ADR cells in the same test system (Figure 4B). Also, the recombinant MVP could be detected in similar Western blot experiments (not shown).

In immunoprecipitation experiments, the anti-MVP Fab was unable to precipitate the MVP protein from either *MVP*-overexpressing GLC4 ADR cells or recombinant MVP in solution (not shown). These results indicate that MVP-φ4 is reactive to a linear or more denatured epitope of the MVP molecule. Of note, the LRP-56 mouse Mab is reactive to a more native epitope, judged from its reactivity in the immunoprecipitation experiments (see Figure 4B), and its inability to react with MVP in Western blots. Also, LRP-56 is unreactive with recombinant MVP coated in ELISA plates (not shown).

### BIAcore analysis of anti-MVP Mabs and MVP-*φ*4

**Figure 5:**
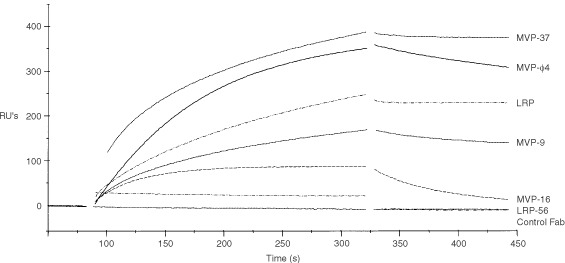
BIAcore analysis of the anti-MVP reagents. The off-rate of several murine anti-MVP Mabs and MVP-φ4 was determined by surface plasmon resonance on a BIAcore 2000 instrument, with recombinant GST-MVP immobilised on the flow cell of a CM-5 sensorchip. The control Fab and LRP-56 Mab (and LMR-5, not shown) were un-reactive in this system. The other murine Mabs MVP-9, MVP-16, MVP-37, LRP and the MVP-φ4 human Fab showed differential reactivity to the MVP molecule immobilised on the chip.

The off-rates of several murine anti-MVP Mabs and MVP-φ4 were determined by surface plasmon resonance on a BIAcore 2000 instrument, with recombinant GST–MVP immobilised on the flow cell of a CM-5 sensorchip, giving a surface of 2000 resonance units. As anticipated, the LRP-56 and LMR-5 Mabs were unreactive with MVP presented in this way, but the other available (bivalent) murine Mabs MVP-9, MVP-16, MVP-37, LRP were reactive with the MVP molecule immobilised on the chip (Figure 5

). These, and the monovalent MVP-φ4 human Fab, gave off-rates of 1.3×10^−3^, 1.4×10^−2^, 1.3×10^−4^, 5.2×10^−5^, and 1.3×10^−3^, s^−1^, respectively.

### Reactivity of anti-MVP Fab on cytospins and tissue sections

**Figure 6:**
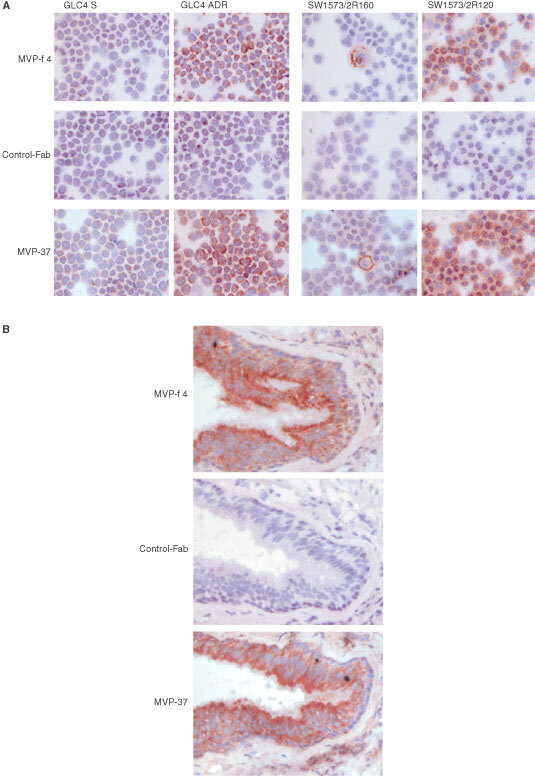
Staining results for MVP in a panel of cell lines with known presence or absence of MVP (**A**) and in frozen sections of normal human lung (**B**). Preparations were pretreated using the guanidine method (see Materials and methods) and stained with MVP-φ4, control Fab or MVP-37 murine Mab. Biotinylated rabbit-anti-mouse and HRP-labelled streptavidin were used as secondary reagents for the mouse Mab. For detection of the human Fabs, 9E10 Mab and HRP-labelled rabbit-anti-mouse serum was used. Colour development was with AEC/H_2_O_2._ Both the anti-MVP Fab MVP-φ4 and the murine Mab MVP-37 show, as expected, the presence of MVP in the GLC4 ADR cells, the SW1573/2R120 cells and in a characteristic low percentage of cells in the MDR1 P-gp positive SW1573/2R160 cells (**A**). Furthermore, MVP-φ4 and MVP-37 show the presence of MVP in the epithelial cells of the bronchi of the lung (**B**).

A panel of cell lines with known presence or absence of MVP was stained with MVP-φ4. MVP-φ4 was only very moderately reactive on preparations of cell lines fixed with 100% acetone. However, using the guanidine fixation method (see Materials and methods), which facilitates detection of more denatured epitopes, the anti-MVP Fab showed the presence of MVP in GLC4 ADR cells, SW1573/2R120 cells and in an earlier identified small subpopulation of cells of the MDR1 P-gp positive SW1573/2R160 cell line (Figure 6A

). Essentially similar results were obtained in control stainings with MVP-37, using the same treatment, or with LRP-56 Mab on acetone fixed cytospins. Further confirmation of the specificity of the anti-MVP Fab was obtained in studying human tissue staining. Using guanidine-treated, frozen sections of human lung tissue, MVP-φ4 was found to be reactive with MVP in the epithelial cells of the bronchi (Figure 6B). Again, comparable staining results were seen in the control stainings with MVP-37 and LRP-56.

## DISCUSSION

For the detection of MVP and transmembrane transporter molecules mediating MDR several conventionally produced murine Mabs have become available. The phage display method allows selection of human antibody fragments, which are expected to elicit less or no immune responses when administered in humans. Using this new technology various human antibodies have now been generated, and some of these have already entered phase III clinical applications (Holliger and Hoogenboom, 1998; Vaughan *et al*, 1998). Up till now, for none of the molecules associated with MDR these type of human antibodies have been generated. Only for MDR1 P-gp humanised versions of the monoclonal antibodies MRK16 (Hamada *et al*, 1990) and MRK17 (Ariyoshi *et al*, 1992) have been constructed. Also, the phage display method has been used successfully to identify peptide epitopes of MDR1 P-gp Mabs (Poloni *et al*, 1995a,b) and peptides mimicking the drug-binding activity of P-gp (Popkov *et al*, 1998). The present selection of a human anti-MVP reactive Fab by the phage display method is, therefore, the first report on truly human antibodies in this field.

During the selection steps of the anti-MVP Fab, successive selection rounds yielded increasing numbers of MVP-specific Fabs. However, *Bst*NI fingerprint and sequence analyses showed that eventually only one type of Fab was isolated. The selection of just one type of Fab may have resulted from the way the selections were performed, causing loss of low affinity phages, e.g. due to low amounts of antigen for selection and high stringency conditions at the elution steps. Also, utilising recombinant MVP as the selection antigen, that may not have full native conformation, and the apparent presence of selection-dominant epitopes in most antigens (Hoogenboom *et al*, 1999) may have influenced the outcome of the selections. On the other hand, although the presently used library has been successfully applied for isolating Fabs against rare antigens and self antigens (de Haard *et al*, 1999), it cannot be excluded that these results still reflect library limitations.

In any case, the isolated MVP-φ4 Fab performed very well in several immunohistochemical and Western blot applications. The immunohistochemical results were optimal when the cytospins and tissues were pretreated with denaturing guanidine/HCl solution. These results, and the unreactivity of the Fab in immunoprecipitation experiments, suggest its reactivity with a linear epitope of the MVP molecule. Of note, both LRP-56 and LMR-5 Mabs are likely to react with more native epitopes, as judged from their good performance in immunoprecipitation experiments, in the absence of Western blot reactivity. Interestingly, BIAcore analyses showed that the MVP-φ4 Fab, as a monovalent fragment, has a relatively slow off-rate (1.3×10^−3^, s^−1^), as frequently observed with conventional antibodies generated from a secondary immune response. Indeed, the MVP-φ4 Fab off-rate was within the range observed for other (bivalent) murine anti-MVP Mabs.

The present identification of an MVP-specific Fab may allow for the construction of plasmids for functional knock-out purposes. The cDNA encoding the Fab can be transferred to an eukaryotic expression plasmid which may also provide a fusion between the Fab and an endoplasmatic reticulum retention signal. Transfection of this new construct into MVP-positive tumour cells then might lead to blockage of the MVP protein, e.g. due to binding of the Fab to the epitope during protein synthesis, and thus interfere with vault assembly and/or function. Recently, the expression of only the MVP molecule in Sf9 insect cells was shown to be sufficient for the assembly of vault-like particles (Stephen *et al*, 2001). Targeting this critical vault component, therefore, is very likely to result in the knock-out of functional vault particles. Analogous applications of ‘intrabodies’ have already been described for e.g. *erb*B-2 (Deshane *et al*, 1994), Ras (Lener *et al*, 2000), huntingtin (LeCerf *et al*, 2001), as well as the anti-P-gp directed C219 ScFv (Heike *et al*, 2001).

In conclusion, a human Fab fragment reactive to MVP, named MVP-φ4, was isolated by phage display technology. This Fab may become a valuable tool in studying the contribution of MVP and vaults to normal physiology and MDR.
